# Behavioural predictability in chickens in response to anxiogenic stimuli is influenced by maternal corticosterone levels during egg formation

**DOI:** 10.1038/s41598-025-19948-x

**Published:** 2025-09-23

**Authors:** Diego Stingo-Hirmas, Lars Rönnegård, Felipe Cunha, Dominic Wright, Rie Henriksen

**Affiliations:** 1 Department of Physics, Chemistry and Biology (IFM), Linköping University, Linköping, Sweden; 2 School of Information and Engineering, Dalarna University, Falun, Sweden; 3 Department of Biosciences, Swedish University of Agricultural Sciences, Uppsala, Sweden

**Keywords:** Prenatal maternal stress, Behavioural plasticity, Anxiety, Predictability, Double hierarchical generalised linear models, Ecology, Ecology, Neuroscience, Psychology, Psychology, Zoology

## Abstract

Across species, prenatal maternal stress has been shown to create heterogeneity in behavioural phenotypes. Research has recently highlighted that individuals vary in how predictable they are in their behavioural responses. This within-individual variation in behaviour is likely to be of biological importance, since individuals interact with the world not only through their mean behavioural phenotype, but also through their full range of behavioural variation. Yet, the underlying mechanisms that create and constrain between-individual variation in behavioural predictability remain largely unexplored. Here, we estimate whether experimental elevation of maternal corticosterone during egg laying (to model prenatal maternal stress) can cause variation in behavioural predictability in a population of chickens. Offspring’s behavioural predictability was quantified by testing them repeatedly (16 times) in a standard anxiety test (open-field test). Elevated maternal corticosterone resulted in less anxious and more predictable offspring compared to control offspring. These findings provide the first evidence that maternal corticosterone levels, via prenatal pathways, may influence multi-hierarchical behavioural plasticity by affecting both the magnitude and the predictability of behavioural responses. These results not only expand our current knowledge about the ways maternal stress can affect offspring’s behavioural phenotypes but also suggest a possible proximate mechanism underlying within-population variation in individual behavioural predictability.

**Supplementary Information:**

The online version contains supplementary material available at 10.1038/s41598-025-19948-x.

## Introduction

Prenatal maternal stress occurs when physiological changes in a female, due to stress exposure, lead to alterations in the prenatal environment she provides for her offspring^1,2^. A growing body of studies has linked this kind of early life stress to altered behavioural responses in the offspring, especially to anxiogenic stimuli (for review see^3–5^). Although prenatal maternal stress has often been equated to increased anxiety in offspring, effects vary between studies and individuals, making conclusions about proximate mechanisms and ultimate consequences difficult^3,5^. While the strength and duration of the stress exposure, as well as genetic factors, are likely to underlie much of this variation^6,7^, how behavioural phenotypes are assessed could also contribute to inconsistencies between studies^8^. Comparisons between individuals in prenatal stress research often rely on single measurements of behavioural traits^3,5,9^. Recent studies have highlighted that some individuals are consistently more predictable than others and that the behaviour of a given individual is better expressed by a distribution of values that is unique to that individual^10^. Even after statistically controlling for individual differences in responses to known gradients (such as habituation), substantial within-individual variation remains^8,11^. This unexplained within-individual variance, called residual intraindividual variation (rIIV), or residual predictability^12^, often amounts to the largest component of behavioural variation within a population^13,14^, yet its significance remains largely unexplained. This is potentially problematic since variation in behavioural predictability between individuals can render single measurements of labile traits, such as behaviour, unreliable as indicators of stress effects^9^. If prenatal maternal stress, through alterations to the prenatal environment, affects behavioural predictability, this would imply that single measurements of behaviour are insufficient to capture the full effect of prenatal maternal stress and might even mask effects of this type of early life stress on average anxiety.

Since individuals interact with the world not only through their ‘average’ behavioural phenotype, but also through their extremes, it has been suggested that behavioural predictability may represent different ways to facilitate learning, social interactions, or avoid predation^15–18^. The pill bug, for example, becomes less predictable in risk-taking behaviour in unfamiliar, rather than familiar environments^19^. While hermit crabs, on average, exhibited higher latency to emerge from their shell and lower predictability in their behaviour when exposed to predator cues^15^, which may be a strategy to improve survival by making their responses less easily anticipated by predators. Potential effects of prenatal maternal stress on predictability might therefore also inform about possible adaptive or maladaptive programming caused by this type of early life stress. While phenotypic responses to prenatal maternal stress are often viewed as unavoidable negative outcomes, the possibility that it prepares the offspring for a stressful environment has been proposed^3^. In that case, we might expect prenatally stressed individuals to be more anxious and unpredictable in their behavioural response to anxiogenic stimuli. Studies on the effects of reduced fitness on behavioural predictability indicate that experimental suppression of the immune system makes individuals more predictable in their behaviour, and suggest that these effects might be linked to lower energy reserves, creating a more conservative behavioural profile^20,21^. Increased behavioural predictability accompanied by changes to mean behavioural anxiety might therefore reflect a maladaptive consequence of prenatal maternal stress on the individual.

In this study, we aimed to investigate whether prenatal maternal stress effects are multi-hierarchical, affecting not only the magnitude of behavioural responses in an anxiogenic test but also how predictable this magnitude is across multiple observations. To see if behavioural predictability represents an important axis of consistent behavioural variation that can be influenced by both present and past stress. To this end, we artificially elevated maternal plasma corticosterone (Cort) during egg laying in female chickens. Cort is the main glucocorticoid in birds and is responsible for mobilising energy during situations of perceived and real threats, leading to alterations in the bird’s physiology and thereby the prenatal environment she provides for her offspring^22^. Chickens, like other birds, develop outside the mother’s body in an egg that has been produced within a short time window, facilitating an easier correlation between the mother’s Cort treatment and the offspring’s prenatal environment and behavioural phenotype^23,24^. We have previously shown that chickens express large intra-individual behavioural variation in response to anxiety-inducing test situations^25,26^ and that this intra-individual variability in behaviour has a direct genetic basis that is largely unique compared to the genetic architecture for the standard mean behavioural measures it was based on^25^. By linking maternal Cort treatment during egg formation with variation in the offspring’s behavioural predictability and overall anxiety, we aim to gain more knowledge about the maintenance of heterogeneity in residual behavioural predictability within a population, and thereby its biological significance.

## Methods

### Animal rearing and corticosterone implants

Females (n = 24) and males (n = 24) from an advanced intercross (F20) of red junglefowl and domestic layer chickens (white leghorn) were paired for breeding in individual cages (92.5 × 57.5 × 65.5 cm) equipped with perches and provided with food and water ad libitum. After four days, the males were removed from the breeding cages. On the following day, all hens were administered 1% lidocaine as local anaesthesia, 30 min prior to the procedure. Hens received a subcutaneous implantation with either corticosterone slow-release pellets (n = 15) or placebo pellets (n = 9) via a small incision on their right flank, which was then closed with two stitches. The ratio between Cort- and placebo-implanted hens was chosen to counter an expected reduction in both egg production and viability caused by the treatment, as glucocorticoids are linked to a decrease in reproductive abilities in multiple species^27–30^. The pellets were obtained from Innovative Research of America (Sarasota, FL, USA) and were designed to release 7.5 mg of corticosterone over a 60-day period. The dose was selected based on higher doses leading to egg-laying cessation in other females from this population. Eggs were collected daily from each hen and immediately marked with the date of laying and family ID using a graphite pencil. On the same day they were laid, eggs were weighed to the nearest 0.01 g and stored at 14 °C in a temperature-controlled storage cabinet equipped with electric egg turning trays. Eggs were placed with their narrow end down and stored until day 12 of egg collection. Storing eggs under these controlled conditions minimises potential effects on viability and allows for delayed incubation so that embryonic development is synchronised across individuals^31–34^.

Previous analysis of eggs laid by similar Cort-implanted female chickens by^23^ showed a significant effect of Cort on egg mass and hormone concentration from day 4 post-implantation, demonstrating that it takes around 4 days for Cort implantation to significantly change the prenatal environment (the egg). This is supported by the fact that although chickens lay an egg every 24 to 26 h, primordial follicles’ terminal differentiation into pre-ovulatory follicles takes 4 to 6 days to occur, and another 24 h pass before being dropped into the infundibulum^35–37^. We used a lower dose than^23^, and therefore used egg mass as a proxy to determine if the prenatal environment (the egg) was also affected by day 4 post-implantation in our study. Maternal treatment had no effect on egg weight on days 1–3 (Welch two-sample t-test: t(18.8) = 1.3, p = 0.21), but eggs from corticosterone-treated mothers were significantly lighter between days 4–12 post-implantation (t(44) = −4.27, p = 0.0001) (see supplementary information Figure S1). Therefore, only eggs laid between days 4 and 12 post-implantation were included in this study as a way to ensure there was enough time during egg formation for treatment effects to reach the egg.

After the 12 days of collection, all selected eggs were placed in a single incubator (25-I HLC; Massalles Europe S. L., Barcelona, Spain) set at 37.8 °C and 55% relative humidity (RH) for 19 days, where they were turned automatically every 6 h by approximately 90°. Two days before the expected hatching date, eggs were transferred to a hatcher (25-N HLC; Massalles Europe S. L., Barcelona, Spain) and placed in trays with individual separators. Each separator was labelled with the same family ID and lay date as marked on the corresponding egg, ensuring that chicks could be reliably traced back to their original egg and family. The hatcher was maintained at 37.5 °C and 65% RH until hatching at day 21. Upon hatching, chicks were immediately weighed and wing-tagged for individual identification.

A total of 54 offspring hatched: 25 Control females, 29 Control males, 8 Cort males, and 9 Cort females, originating from 6 corticosterone-treated mothers and 9 placebo-treated mothers. This imbalance in offspring numbers between treatments was anticipated and reflects the known effects of elevated glucocorticoids on reproduction. Despite implanting more Cort-treated hens (n = 15) than placebo hens (n = 9) to compensate, Cort exposure still led to both reduced egg production and lower hatching success, consistent with previous findings that elevated glucocorticoids negatively affect reproductive output and embryo viability in birds and other species^27,28^. All hatchlings from both Cort- and placebo-implanted females were housed together in collective indoor pens (1 × 1x2 m) with perches and a heating lamp, with unrestricted access to food and water. At 7 weeks of age, they were moved to two large collective pens (4 × 4x4m) with access to an outdoor enclosure (4 × 3x4m) and divided by sex.

### Open-field behaviour test

Offspring underwent an open-field behavioural test once per day for eight days across two consecutive weeks, assessed at two life stages: both early in life, starting at one week of age (early-age) and once they had reached sexual maturity after 5 months of age (late-age). The tests were carried out once a day, over four consecutive days, then left for a 3-day weekend pause, and repeated again during the second week. Each offspring was tested a total of 16 times in the open-field test: 8 times early in life and 8 times after sexual maturity. During early-age testing, individuals were placed in an open-field arena measuring 117 cm by 80 cm with a rubber floor (see supplementary information Figure S2 A). Individuals were placed in the arena in darkness, via a small hatch in the corner of the arena. For the late-age tests, the arena measured 293 cm by 285 cm and the floor was covered in wood shavings (see supplementary information Figure S2 B), with the procedure otherwise being identical to the early-stage test. When the tests started, the lights were turned on, and the individuals were video recorded for five minutes. Testing at both early and late ages was always performed between 9:00 AM and 2:00 PM, with animals assessed in a randomised sequence, and the arenas were cleaned and reset after each test. The offspring’s position in each video frame was tracked using an AI object detection model pre-trained on 2451 labelled images extracted from the same open-field videos, using the YOLOv7 object-detection algorithm^38^.

For analysis, two regions of interest (edge and inner zone) were defined: The edge corresponding to the space 20 cm immediately around the arena edges during early-age tests, and 61 cm during late-age tests. Two behaviours were tracked during the open-field test: 1) total distance travelled measured in centimetres and 2) total time spent at the edge (measured in seconds). These two scores were analysed in R^39^, and tracked movement was quantified using modified code from Sturman et al.^40^. Distance travelled and time at the edge have previously been proposed to measure distinct aspects of animal behaviour during the open-field test in both mammals^41^ and birds^42–44^. Movement activity during the open-field test is commonly associated with an exploratory response^45–47^, while proximity to the edge of the arena is considered representative of wariness or anxiety^48,49^.

### Statistical analysis

Behavioural response scores were transformed by applying Ordered Quantiles (ORQ) normalisation, using the “BestNormalize” package in R^50,51^. Behavioural predictability was quantified statistically using double hierarchical generalised linear models (DHGLM^52,53^;). This extension of a mixed model allows for simultaneous modelling of differences in the average and the residual variance by fitting variables for both a ‘mean’ and a ‘dispersion’ section of the model. While the former section models the response variable’s mean values, the latter estimates the effects of the response variable’s dispersion. Thus, fixed effect coefficients of the dispersion model indicate whether a given factor or gradient increases or decreases the response’s variance. The model uses extended quasi-likelihood for estimation^54^, accommodating heteroscedasticity and providing more robust estimation when data exhibit unequal variances or heavy-tailed distributions^53,55^. Finally, DHGLMs allow the inclusion of random effects over the response variable in both sections of the model. We include a random effect of offspring-ID in the dispersion model, which allows estimation of differences in within-individual residual variance, rIIV^11,56^, interpreted as each individual’s effect on the dispersion of the response variable. Higher values of rIIV indicate that an individual produces a wider range of behavioural responses relative to the average behavioural variance in the population; hence, it behaves less predictably. In contrast, individuals with low rIIV only express a narrow range of behavioural values and are therefore considered to behave more predictably.

DHGLMs were built in R using a modified version of the functions provided in the “hglm” package^54,57^. Fixed effects included in the mean and dispersion model were ‘testing week’ (week 1 or 2), ‘day of testing’ (days 1 to 4), ‘age at which they were tested’, ‘sex’, and ‘treatment’ (either placebo or CORT maternal implants). An interaction between the day and week of each test was included to account for habituation effects across the two testing weeks. No significant interactions between sex and treatment or between age and treatment were observed for any behavioural responses (p > 0.1) and were therefore excluded from the final models (see models 1.1 and 1.2). Egg mass was not included as a predictor in the final models, as it is strongly influenced by treatment and would not provide independent explanatory power. Individual random effects were grouped by age for both the mean and the dispersion models, meaning each individual had a different estimated random effect on the mean and predictability for early and late rounds of open-field testing, respectively. Finally, effects caused by similarities between siblings were accounted for by including a family identifier as an additional random effect on the intercept (FamilyID). The model can then be represented as:

1.1 $$\begin{array}{c}y=Day+Week+Day:Week+Sex+Treatment+Age+re\left(ID:Age+FamilyID\right)+e\end{array}$$

The residuals, $e$, are normally distributed with the residual variance, ${\sigma }_{e}^{2}$, being modelled by a dispersion model:

1.2 $$\begin{array}{c}\text{log}\left({\sigma }_{e}^{2}\right)=Day+Week+Day:Week+Sex+Treatment+re\left(ID:Age+FamilyID\right)\end{array}$$

where the first Eq. (1.1) models the mean values of the response variable *y* (a vector of the scores recorded, either distance travelled or time at the edge), and the second Eq. (1.2) models the residual deviations from the prediction (*y*). The random effect (*re*) of individuals is grouped by early and late age of test rounds in the mean and the dispersion part of the model. Hence, two variance components per individual are estimated in the dispersion part of the model, which measures rIIV at the two ages. A single maternal random effect was included that further groups observations per family. Models were fitted separately for both distance travelled and time at the edge during the 5-min session. Finally, post-hoc analyses were performed using Spearman’s rank correlation tests to assess the relationship between individual estimated predictability at early and late ages, as well as the relationship between individual effects on the mean and on the dispersion.

## Results

**Fig. 1:**
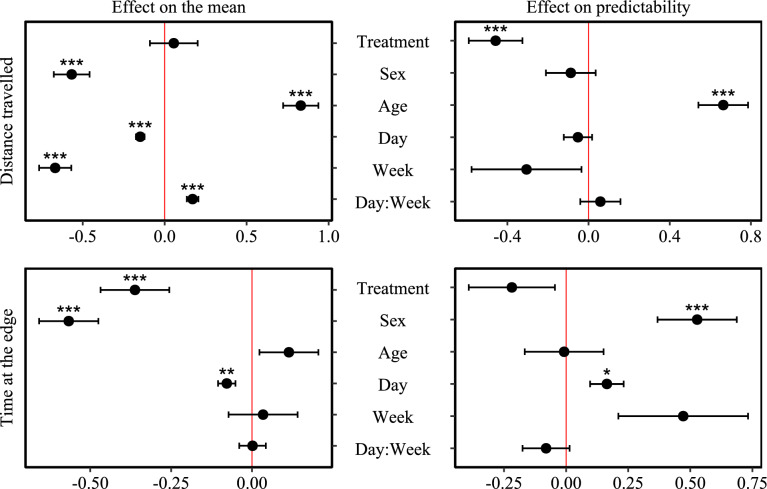
DHGLM fit estimates effects on the mean and dispersion models for Distance travelled and Time at the edge for chickens tested in open field test. Effects of treatment, sex, age and week are presented in reference to Placebo, Female, Early-age and Week 1, respectively. (Asterisks note estimate significance where * = p < 0.05, ** = p < 0.01, and *** = p < 0.001).

The estimated parameters of the fitted models for the two behavioural traits measured (distance travelled and time at the edge) in the open-field test are summarised in the supplementary information table S1 and table S1 respectively (see also Fig. 1).

### Treatment effects on mean response and predictability

Treatment effects on the offspring’s behaviour (time spent at the edge and distance travelled) in the open-field test and the behavioural predictability of their response across testing days were measured by the fixed effects estimates in the mean part of the model [Eq. 1.1] and the dispersion part of the model [Eq. 1.2] respectively.

Offspring of Cort-treated mothers spent on average significantly less time at the edge of the open field arena than control offspring (β = −0.36 ± 0.1; p < 0.001), suggesting that they were less anxious during the test. Treatment did not affect the predictability of time spent at the edge (β = −0.22 ± 0.17; p = 0.21). For distance travelled, the mean was unaffected by maternal treatment (β = 0.06 ± 0.15; p = 0.7), but it significantly increased behavioural predictability, with Cort-offspring showing less variability across trials than controls (i.e., negative effect on dispersion for distance travelled, β = −0.46 ± 0.13; p < 0.001).

### Effects of age and sex

Offspring’s distance travelled increased significantly with age (β = 0.83 ± 0.11; p < 0.001), and individuals were less predictable across trials compared to early-age (β = 0.66 ± 0.12; p < 0.001). Age did not affect time spent at the edge (p = 0.21) or how predictable individuals were in this behaviour (p = 0.96). Sex had a strong effect on the individual’s behaviour, with males travelling less (β = −0.57 ± 0.11; p < 0.001) and spending significantly less time at the edge (β = −0.57 ± 0.09; p < 0.001) than females. Males were also less predictable when measuring time spent at the edge (β = 0.53 ± 0.16; p < 0.001), indicating that although they spent less time at the edge of the open-field, their behaviour varied more across trials.

### Variation in mean and predictability across testing trials and individuals

Irrespective of treatment, the offspring reduced both their distance travelled and time spent at the edge across test days (β = −0.15 ± 0.03; p < 0.001 and β = −0.08 ± 0.03; p = 0.004, respectively). For distance travelled, this habituation effect carried over into the second week of testing: a large week effect (β = −0.66 ± 0.1; p < 0.001) combined with a small, positive day × week interaction (β = 0.17 ± 0.04; p < 0.001) indicates that although the decline in distance travelled was maintained, the rate of decline slowed during the second week. In contrast, for time spent at the edge, no significant differences were observed between weeks (p > 0.7), suggesting that the habituation effect did not carry over but instead “reset” when testing resumed during the second week. The predictability of time at the edge decreased across trials (β = 0.16 ± 0.07, p = 0.015), whereas no such effect was found on the predictability of distance travelled (p = 0.45).

### Post-hoc analyses

Across ages, individuals travelling greater distances tended to show lower behavioural variability and thus higher predictability (Spearman’s rho = −0.26, p = 0.007). This relationship was strongest at late-age tests (rho = −0.36, p = 0.008). For time spent at the edge, no overall association with predictability was found, but a significant negative correlation emerged at late age (rho = −0.30, p = 0.030). In late age tests, individuals who travelled greater distances also spent more time at the edge (rho = 0.29, p = 0.037), suggesting a potential behavioural syndrome at this stage. However, no significant correlations were found between individual predictability estimates for distance travelled and time spent at the edge at any age (all |rho|< 0.22, all p > 0.1), indicating that variability in behaviour is largely trait-specific. Finally, neither mean behavioural tendencies nor predictability scores were consistent between early and late life for either trait (all |rho|< 0.25, all p > 0.05), suggesting that both the individual magnitude of behavioural responses and behavioural predictability measured during early life are not maintained into adulthood.

## Discussion

Maternal Cort treatment affected both offspring’s anxiety-related behaviour in the open-field test as well as how predictable they were in their behavioural response, demonstrating for the first time that prenatal maternal Cort levels can influence behavioural predictability and generate heterogeneity in residual intra-individual variation within a population. On average, offspring of Cort-implanted mothers spend less time at the edge of the open field arena, suggesting they were less anxious than control offspring. This is contrary to the general perception that prenatal maternal stress leads to increased anxiety^2,5,58^, but similar to a previous study on chickens^24^, where Cort-implanted mothers also produced less anxious offspring, as well as other studies on rodents^59–62^. Reflecting the often-contradicting findings of prenatal maternal stress on offspring’s anxiogenic behaviour, as mentioned in the introduction. While we hypothesised that variation in predictability could explain some of these inconsistencies by masking effects of prenatal maternal stress on mean anxiety this is not supported by our finding that maternal Cort elevation affected mean anxiety in one trait and predictability in another trait. The effects of maternal Cort treatment on predictability were only seen when measuring total distance travelled in the open field, but did not affect the offspring’s predictability when measuring time at the edge, suggesting that maternal stress effects on predictability could be trait specific. Movement and time spent at the edge of the open field have previously been shown to have separate underlying genetic architecture^63–65^, illustrating that selection can act on each trait separately. Effects of prenatal maternal stress on offspring anxiety in the open field have previously been shown to be trait-specific. A study by Tazumi et al.^66^, for example, found that prenatally stressed rats didn’t differ from control individuals in their locomotor activity but had a significantly higher startle response, while van den Hove et al.^62^ found no differences in locomotor activity, but did find significant differences in time at the edge in prenatally stressed rats. Several other studies on prenatal maternal stress (either via experimental elevation of maternal plasma cort levels or by exposing the mother to stressful events) reported no effect on average movement activity in the offspring of birds^24,67^ and mammals^59,68–70^, similar to our finding, yet none of these studies report on the predictability of the offspring’s behaviour, which we observe to be highly affected by maternal Cort levels. The fact that maternal Cort levels during egg formation can influence behavioural predictability in a trait-specific manner suggests that predictability, like other personality traits^71^, might have a clear genetic basis that is unique compared to the genetic architecture for the standard behavioural measures they are based on. Predictability has been observed to possess certain levels of heritability and covariation across behavioural scores^25,72,73^ although identification of the molecular basis underlying behavioural predictability is still largely missing (but see^25^).

Overall, our findings suggest that the offspring of Cort-treated mothers were less affected by the environment, being less anxious in the open field test and showing less fluctuation in behaviour from one trial to the next by being more predictable. These findings don’t fit the general literature that being more unpredictable prepares you for a precarious environment, but could indicate that elevated maternal cort levels during egg laying create more robust individuals when faced with potentially stressful situations.

Generally, effects of prenatal maternal stress are seen as an unavoidable negative outcome due to a suboptimal prenatal environment^74^. To our knowledge, only a handful of studies^20,21,75^ have looked at behavioural predictability in fitness-challenged individuals. Winter and colleagues^21^ found that immune-impaired grasshoppers were more predictable than control individuals when measuring their jump distance, which the authors suggested could be due to lower energy availability acting as a limiting factor in the variability of jump distances*.* Support for a correlation between energy availability and predictability was also observed in a study by Klaassen and colleagues^75^, where planaria exposed to the carcinogen cadmium had lower activity levels and higher predictability. The authors suggested that control individuals can perform “short bursts of activity” that are difficult to maintain over an extended time, thus increasing both their activity’s mean and variance. In our results, prenatally stressed individuals did not show lower activity levels but did have higher predictability, just like the more fitness-challenged grasshoppers^21^ and planaria^75^. Previous research has associated variation in behavioural predictability with key energetic traits^10,76^. For example, laboratory mouse lines with greater aerobic scope (i.e., difference between maximum and minimum metabolic rate) were shown to be more unpredictable in their behaviour than lines with smaller aerobic scope^77^. The authors hypothesised that individuals with greater aerobic scope may have an increased capacity to express behavioural variation, and thus will be more unpredictable in their behaviour. Since prenatal maternal stress is known to affect various metabolic parameters across species^78^, it’s not unlikely that the lower predictability in offspring of Cort-treated mothers is due to the side effects of the mother’s cort treatment on the offspring’s metabolic system. Indicating that increased behavioural predictability signals reduced fitness in offspring of Cort-treated mothers.

Similar to our study, the environmental effects on predictability reported above were trait-specific, suggesting modularity in predictability between traits and the degree to which the environment affects this level of intra-individual variation. Additionally, Beyts and colleagues^20^ found, using a split brood design, that raising African clawed frog tadpoles (*Xenopus laevis*) on high or low food availability affected the tadpoles’ behavioural predictability when measuring swimming distance, but that the degree and direction of predictability were dependent on whether the tadpoles were tested in a familiar or unfamiliar environment. Their results suggest that behavioural predictability is a highly plastic trait, which can be influenced by environmental changes throughout early life. The process by which the environment could modulate intraindividual variation in behavioural responses is still uncertain. Under the incomplete model hypothesis (suggested by^8^), maternal Cort levels could modulate the predictability of exploratory behaviour in the offspring by altering the linearity of the behavioural responses across repeated trials, or by affecting an individual’s response to an unaccounted environmental factor (i.e., multidimensional reaction norms, see^8,79^). If prenatal maternal stress affects an individual’s sensitivity to the environment, even in a controlled test, a prenatally stressed individual could show lower sensitivity to external input and exhibit a more consistent behavioural response across repeated trials. Prenatal maternal stress could also interact with the occurrence of random residual predictability by affecting an intrinsic characteristic of an individual’s expression of exploratory behaviour, where, if such a process is costly, suppression of adaptive predictability could lead to a reduction in variability of the responses observed across tests for treated individuals, as seen in the aforementioned studies^20,21,75^.

The offspring’s behavioural predictability in distance travelled decreased with age, regardless of maternal treatment, indicating that individuals became less consistent in their behavioural responses after reaching sexual maturity. Moreover, no significant correlations were observed between early- and late-age individual effects on either the mean or the dispersion of behaviour, suggesting that behavioural tendencies and predictability measured in early life are not maintained into adulthood. Personality traits based on anxiety tests in chickens (red Junglefowl) have previously been shown not to stabilise before sexual maturity^80^, suggesting that predictability shows a similar ontogeny as other personality traits. However, given the size differences between the offspring at one week old versus sexual maturity, we cannot exclude that this age effect is purely due to variation in distance travelled between chicks and adult chickens in an open field arena. This is further supported by the lack of an observed effect of age on the time spent at the edge of the open field, both regarding the mean and dispersion models, which can be seen as supporting the comparability between tests at both ages.

Effects of maternal Cort treatment were not dependent on the sex of the offspring, but males, regardless of treatment, spent less time at the edge than females, moved less during the test, and were less predictable when measuring their time at the edge. Sex differences have been found in other studies on the predictability of behavioural traits like exploration^81^, sociability^82^, and boldness^83^ across species, suggesting that behavioural predictability depends on differences in selection pressures experienced by each sex^18^. Sexual selection driven by female mate choice has been proposed as one of the main driving forces^84^, as it has been observed that sexual selection increases male behavioural predictability in Drosophila^85^ and increases attractiveness in cichlid^84^. Predictable individuals might more reliably signal strength by being more consistent and strategic in their behaviour, while unpredictable individuals’ behaviour may prevent opponents from anticipating actions, reducing their likelihood of injury or defeat^9,10,86,87^. This aligns with findings in predator–prey interactions, where unpredictability confers an adaptive advantage^19,88^. Unpredictability may therefore serve as a strategy for weaker individuals facing a challenging situation.

At sexual maturity, our results showed a positive correlation between mean distance travelled and time spent at the edge, suggesting the emergence of a potential behavioural syndrome later in life. However, no significant correlations were found between individual predictability across these two traits, indicating that while mean behavioural tendencies could be linked, variability in behaviour remains largely trait-specific. Although our study indicates that maternal cort elevation during egg formation in chickens leads to less anxious individuals that are more predictable in their behaviour, we cannot assure this behavioural profile to be consistent across other situations and tests. Previous studies have found that individuals’ levels of behavioural predictability are assay-specific when testing individuals across various tests^83^. Nevertheless, our study highlights how standard analytical approaches, where individuals are only tested once in behavioural tests, will likely miss interesting and important components of variation among individuals caused by prenatal maternal stress.

## Conclusions

Our findings demonstrate for the first time that maternal Cort levels during egg formation underlie multi-hierarchical behavioural plasticity in the offspring that hatch from these eggs, by affecting both predictability in their behavioural response to an anxious stimulus as well as their overall level of anxiety. These results not only expand our knowledge about the ways prenatal maternal stress can potentially affect offspring behavioural phenotypes but also suggest a possible proximate mechanism underlying within-population variation in individual behavioural predictability.

## Supplementary Information

Supplementary Information 1.

Supplementary Information 2.

## Data Availability

The datasets generated and analysed during the current study are publicly available through Dryad^89^ (10.5061/dryad.k0p2ngfjr).
