# Supplementary material for: Behavioural predictability in chickens in response to anxiogenic stimuli is influenced by maternal corticosterone levels during egg formation
Source: Sci Rep. 2025 Sep 23;15:32670. doi: 10.1038/s41598-025-19948-x (PMC12457645; doi:10.1038/s41598-025-19948-x)
Supplement: Supplementary file 1 — Supplementary Information 1. [file 41598_2025_19948_MOESM1_ESM.docx]

**Chickens exposed to prenatal maternal stress are more predictable in their**

**behavioural response to anxious stimuli than non-stressed pen-mates**

# **Supplementary information figures and tables**

**Figure S1.** Linear regression of egg mass of corticosterone (red) and placebo (blue) treated mothers. Data includes both viable and non-viable eggs (n = 116). Maternal treatment had no effect on egg weight on days 1-3 (Welch two-sample t-test: t(18.8) = 1.3, p = 0.21), but eggs from corticosterone-treated mothers were significantly lighter between days 4-12 post-implantation (t(44) = -4.27, p = 0.0001).


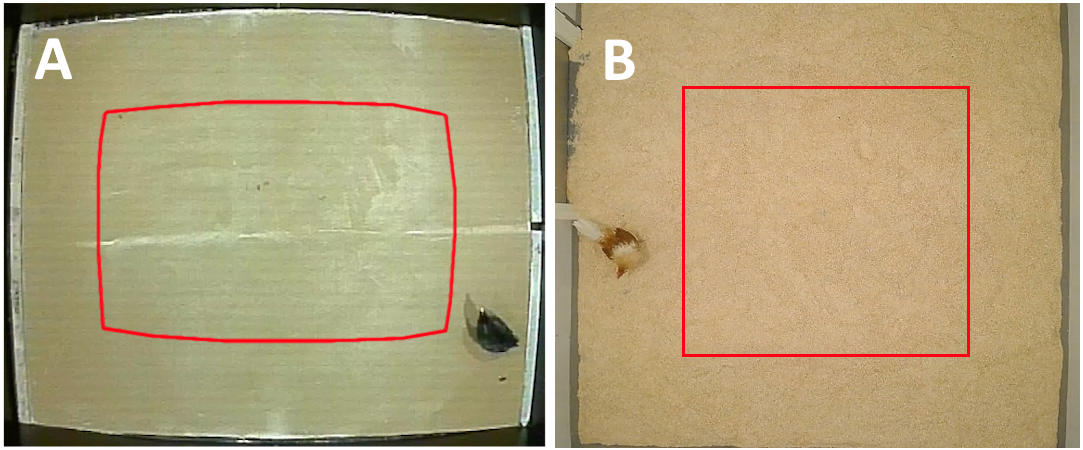


**Figure S2.** Open-field test arenas. Red lines separate the edge from the centre zones defined to estimate the total time spent at the edge of the arena in each test run. A) arena used for early-age tests. B) arena used for late-age tests.

**Table S1.** DHGLM parameter estimates for Distance Travelled (DT) during open field test:

| Variable | Estimate | Std. Error | t-value | p-value |  |
| --- | --- | --- | --- | --- | --- |
| **MEAN MODEL** |  |  |  |  |  |
| (Intercept) | 0.39236 | 0.13417 | 2.924 | 0.00355 | ** |
| Day | -0.14920 | 0.02605 | -5.728 | 1.46e-08 | *** |
| Week - 2 | -0.66809 | 0.09811 | -6.810 | 1.97e-11 | *** |
| Sex - Male | -0.56727 | 0.10938 | -5.186 | 2.74e-07 | *** |
| Age - Late | 0.83022 | 0.10753 | 7.721 | 3.59e-14 | *** |
| Treatment - CORT | 0.05563 | 0.14549 | 0.382 | 0.70231 |  |
| Day:Week2 | 0.17020 | 0.03544 | 4.802 | 1.89e-06 | *** |
| **DISPERSION MODEL** |  |  |  |  |  |
| (Intercept) | -0.93060 | 0.21633 | -4.302 | 1.89e-05 | *** |
| Day | -0.05251 | 0.06965 | -0.754 | 0.45111 |  |
| Week - 2 | -0.30579 | 0.27071 | -1.130 | 0.25897 |  |
| Sex - Male | -0.08766 | 0.12271 | -0.714 | 0.47521 |  |
| Age - Late | 0.66392 | 0.12235 | 5.426 | 7.54e-08 | *** |
| Treatment - CORT | -0.45802 | 0.13224 | -3.463 | 0.00056 | *** |
| Day:Week2 | 0.05830 | 0.09896 | 0.589 | 0.55599 |  |

**Table S2.** DHGLM parameter estimates for Time at the Edge (TE) during open field test:

| Variable | Estimate | Std. Error | t-value | p-value |  |
| --- | --- | --- | --- | --- | --- |
| **MEAN MODEL** |  |  |  |  |  |
| (Intercept) | 0.471836 | 0.110728 | 4.261 | 2.28e-05 | *** |
| Day | -0.077949 | 0.027022 | -2.885 | 0.00403 | ** |
| Week - 2 | 0.034293 | 0.106729 | 0.321 | 0.74806 |  |
| Sex - Male | -0.566598 | 0.091670 | -6.181 | 1.02e-09 | *** |
| Age - Late | 0.114149 | 0.091182 | 1.252 | 0.21099 |  |
| Treatment - CORT | -0.361792 | 0.106310 | -3.403 | 0.00070 | *** |
| Day:Week2 | 0.001749 | 0.041016 | 0.043 | 0.96600 |  |
| **DISPERSION MODEL** |  |  |  |  |  |
| (Intercept) | -1.417520 | 0.234931 | -6.034 | 2.45e-09 | *** |
| Day | 0.164145 | 0.067574 | 2.429 | 0.015356 | * |
| Week - 2 | 0.471513 | 0.261198 | 1.805 | 0.071421 | . |
| Sex - Male | 0.527601 | 0.159442 | 3.309 | 0.000978 | *** |
| Age - Late | -0.007929 | 0.158799 | -0.050 | 0.960188 |  |
| Treatment - CORT | -0.218383 | 0.173449 | -1.259 | 0.208375 |  |
| Day:Week2 | -0.080667 | 0.094886 | -0.850 | 0.395495 |  |
