# Supplementary material for: Behavioural predictability in chickens in response to anxiogenic stimuli is influenced by maternal corticosterone levels during egg formation
Source: Sci Rep. 2025 Sep 23;15:32670. doi: 10.1038/s41598-025-19948-x (PMC12457645; doi:10.1038/s41598-025-19948-x)
Supplement: Supplementary file 2 — Supplementary Information 2. [file 41598_2025_19948_MOESM2_ESM.docx]

**Behavioural predictability in chickens in response to anxiogenic stimuli is influenced by maternal corticosterone levels during egg formation**

# **Supplementary figures and tables**

**Figure S1.** Linear regression of egg mass of corticosterone (red) and placebo (blue) treated mothers. Illustrating changes in egg mass in eggs laid between 1-12 days after pellet implantation. Data includes both viable and non-viable eggs (n = 116).


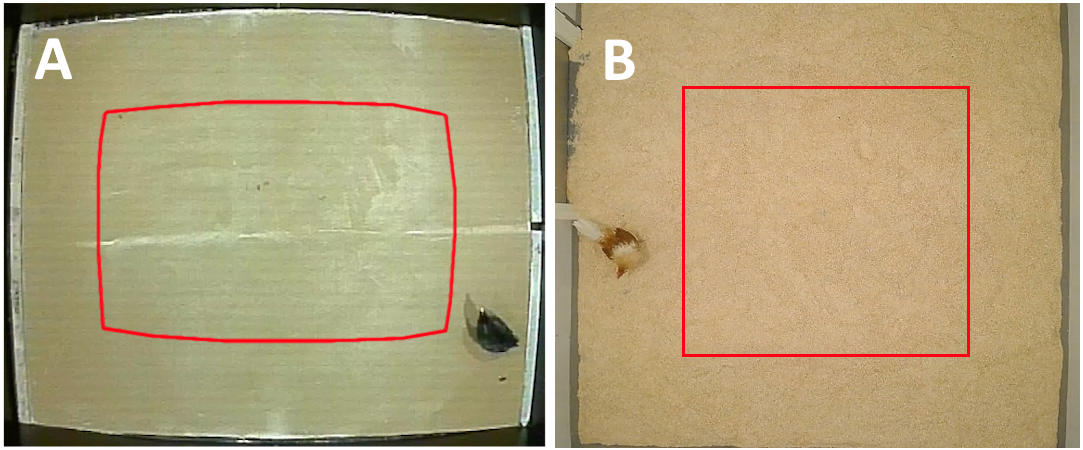


**Figure S2.** Open-field test arenas. Red lines separate the edge from the centre zones defined to estimate the total time spent at the edge of the arena in each test run. A) arena used for early-age tests. B) arena used for late-age tests.

**Table S1.** DHGLM parameter estimates for Distance Travelled (DT) during open field test:

| Variable | Estimate | Std. Error | t-value | p-value |  |
| --- | --- | --- | --- | --- | --- |
| **MEAN MODEL** |  |  |  |  |  |
| (Intercept) | 0.39236 | 0.13417 | 2.924 | 0.00355 | ** |
| Day | -0.14920 | 0.02605 | -5.728 | 1.46e-08 | *** |
| Week - 2 | -0.66809 | 0.09811 | -6.810 | 1.97e-11 | *** |
| Sex - Male | -0.56727 | 0.10938 | -5.186 | 2.74e-07 | *** |
| Age - Late | 0.83022 | 0.10753 | 7.721 | 3.59e-14 | *** |
| Treatment - CORT | 0.05563 | 0.14549 | 0.382 | 0.70231 |  |
| Day:Week2 | 0.17020 | 0.03544 | 4.802 | 1.89e-06 | *** |
| **DISPERSION MODEL** |  |  |  |  |  |
| (Intercept) | -0.93060 | 0.21633 | -4.302 | 1.89e-05 | *** |
| Day | -0.05251 | 0.06965 | -0.754 | 0.45111 |  |
| Week - 2 | -0.30579 | 0.27071 | -1.130 | 0.25897 |  |
| Sex - Male | -0.08766 | 0.12271 | -0.714 | 0.47521 |  |
| Age - Late | 0.66392 | 0.12235 | 5.426 | 7.54e-08 | *** |
| Treatment - CORT | -0.45802 | 0.13224 | -3.463 | 0.00056 | *** |
| Day:Week2 | 0.05830 | 0.09896 | 0.589 | 0.55599 |  |

**Table S2.** DHGLM parameter estimates for Time at the Edge (TE) during open field test:

| Variable | Estimate | Std. Error | t-value | p-value |  |
| --- | --- | --- | --- | --- | --- |
| **MEAN MODEL** |  |  |  |  |  |
| (Intercept) | 0.471836 | 0.110728 | 4.261 | 2.28e-05 | *** |
| Day | -0.077949 | 0.027022 | -2.885 | 0.00403 | ** |
| Week - 2 | 0.034293 | 0.106729 | 0.321 | 0.74806 |  |
| Sex - Male | -0.566598 | 0.091670 | -6.181 | 1.02e-09 | *** |
| Age - Late | 0.114149 | 0.091182 | 1.252 | 0.21099 |  |
| Treatment - CORT | -0.361792 | 0.106310 | -3.403 | 0.00070 | *** |
| Day:Week2 | 0.001749 | 0.041016 | 0.043 | 0.96600 |  |
| **DISPERSION MODEL** |  |  |  |  |  |
| (Intercept) | -1.417520 | 0.234931 | -6.034 | 2.45e-09 | *** |
| Day | 0.164145 | 0.067574 | 2.429 | 0.015356 | * |
| Week - 2 | 0.471513 | 0.261198 | 1.805 | 0.071421 | . |
| Sex - Male | 0.527601 | 0.159442 | 3.309 | 0.000978 | *** |
| Age - Late | -0.007929 | 0.158799 | -0.050 | 0.960188 |  |
| Treatment - CORT | -0.218383 | 0.173449 | -1.259 | 0.208375 |  |
| Day:Week2 | -0.080667 | 0.094886 | -0.850 | 0.395495 |  |
